# *Agrobacterium*-Mediated Transient Gene Expression in Developing *Ricinus communis* Seeds: A First Step in Making the Castor Oil Plant a Chemical Biofactory

**DOI:** 10.3389/fpls.2019.01410

**Published:** 2019-10-30

**Authors:** Alfonso Sánchez-Álvarez, Noemí Ruíz-López, Antonio Javier Moreno-Pérez, Enrique Martínez-Force, Rafael Garcés, Joaquín J. Salas

**Affiliations:** ^1^Department of Biochemistry and Molecular Biology of Plant Products, Instituto de la Grasa (CSIC), Sevilla, Spain; ^2^Facultad de Ciencias, Universidad de Málaga, Málaga, Spain

**Keywords:** *Ricinus communis*, transient expression, promoter, *Lesquerella fendleri*, elongase, acyl-CoA Pool

## Abstract

The castor oil plant represents a promising platform to produce oils with industrial applications. However, its use in biotechnology is limited by the absence of a well-established procedure to transform it, and a poor understanding of gene regulation and promoter use in this species. As such, a method has been developed to express proteins or hairpin-RNA in this plant, a method based on the direct injection of *Agrobacterium* into the developing endosperm of castor oil fruit, enabling different constructs and promoters to be tested. This method produces a high rate of transformation and a good proportion of viable seeds that express reporter genes for up to 20 days after infiltration (DAI). Gene expression under the control of different promoters was tested by quantitative real-time polymerase chain reaction and by directly assaying the activity of the galactouronidase reporter gene, which proved to be strongest when driven by the glycinin promoter. Constructs expressing a fatty acid elongase from *Lesquerella fendleri* were tested, the expression of which provoked an important increase in the lesquerolic acid in the castor oil endosperm at 5 and 10 DAI, although this fatty acid did not accumulate significantly in the final mature seeds. The nature of this response could reflect the poor availability of substrates for this enzyme. In the light of this data, the potential of this technique to test promoters and different constructs in castor oil plants and other oilseeds is discussed.

## Introduction

Castor (*Ricinus comunis*) seed oil differs from other vegetable oils as it accumulates large amounts (up to 90%) of the hydroxylated ricinoleic acid (12-hydroxyl-octadecen-9-oic acid, 18:1-OH) in its triacylglycerols (TAGs). Non-edible, it has largely been used in cosmetics and medicine, and it is considered an important raw material for oleochemistry, particularly as ricinoleic acid confers it a high polarity, viscosity and reactivity (Mutlu and Meier, 2010). Thus, the methyl ester of ricinoleic acid can be cracked to produce undecylenic acid, a precursor of nylon-11, and heptanal, an ingredient of perfumes and essences (Das et al., 1989). It is also a substrate for the production of sebacic acid and 2-octanol, which have similar applications. Furthermore, ricinoleic acid is the precursor of many greases and biolubricants, and it is also used to produce biobased coatings and biofuels (Dwivedi and Sapre, 2002; Berman et al., 2011; Thakur and Karak, 2013).

The metabolism of castor has been of interest to plant biochemists due the capacity of this plant to accumulate high proportions of ricinoleic acid. In castor oil plants, the main extraplastidial modification of fatty acids is the hydroxylation of oleate, which is catalysed by the FAH12 hydroxylase, a membrane-bound enzyme closely related to FAD2 and FAD3 (Van De Loo et al., 1995). This enzyme is very active in developing castor oil seed endosperm and it can insert a hydroxyl group at the delta 12 position of oleate. Nevertheless, the fatty acid composition of castor oil cannot be explained by the activity of this enzyme alone. There is a large body of experimental evidence demonstrating that the synthetic machinery of castor seeds is specialized in the channelling of ricinoleic acid into TAGs, implicating the whole pathway and not just discrete enzymes (Bafor et al., 1991; Kroon et al., 2006; Van Erp et al., 2011; Hu et al., 2012). This biochemical feature make the castor plant a very interesting platform for the production of unusual fatty acids, especially hydroxylated fatty acids of industrial interest. In this regard, the castor plant is also of particular biotechnological interest as it is a productive, non-demanding plant that can grow in many climates, permitting its cultivation as either a tree or an annual crop (Baldwin and Cossar, 2009).

Important advances have been made in the use of castor oil plants as biofactories upon removing the seed toxicity (Sousa et al., 2017). Moreover, a first draft of the plant genome has been presented, revealing the plant’s genetic dotation (Chan et al., 2010). Castor oil mutants with altered fatty acid compositions have also been reported and could form the basis for the future production of other specialized fatty acids in this plant, for example the *OLE1* mutant that contains up to 80% oleic acid and that has a lower ricinoleic acid content (Venegas-Calerón et al., 2016). The production of transgenic castor oil plants has also been reported using a protocol based on the transformation of seed dissected embryos, followed by selection and plant regeneration (Sujatha and Sailaja, 2005; Ahn et al., 2007). However, meristem-based protocols have shown very low transformation efficiency in this plant (0.04%). In this regard, permanent transformation of certain plants is generally a complex process with many drawbacks and consequently, plant physiology, biochemistry and biotechnology research of these plants is often supported by transient gene expression studies.

Transient transformation of plant tissues does not provide permanent integration of the exogenous DNA in the plant progeny. However, these methods make possible an easy and fast testing of new constructs and promoters, which is especially important for plants like castor that are difficult to transform and takes long time to regenerate. Furthermore, these transient expression methods allow making reliable studies of protein location by expression of fluorescence-tagged protein derivatives and the production of specific proteins in specific plant organs. The most extended method for *in vivo* transient transformation involves *Agrobacterium* infiltration, initially developed in *Nicotiana benthamiana* leaves (reviewed in Potrykus, 1991), and since extended to many types of plants and different organs (Wroblewski et al., 2005). Thus, transient expression mediated by *Agrobacterium* injection has been employed extensively in strawberry fruits, representing a powerful tool for gene silencing and a moderate one for protein overexpression (Carvalho et al., 2016). This method has also been used to express proteins of interest in other fruits like tomato (Orzaez et al., 2006) and melon (Han et al., 2015), providing the possibility to produce antibodies and vaccines in an edible plant host. With regard to oilseeds, agroinfiltration efficiently induces transient transformation of detached soybean cotyledons (King et al., 2015), expressing the galactouronidase (GUS) marker introduced into a T-DNA transferred by the bacteria 2-4 days after imbibition (DAI). A similar procedure was also successfully assayed in detached cotyledons from castor oil plants (Chileh et al., 2010) and it was used to test promoters from different 11S globulins strongly expressed in these seeds. Nevertheless, no studies into the engineering of oil synthetic pathways have been tested in this species to date. The oil synthesis pathway is long and complex and the process of oilseed filling usually takes several weeks (8-9 weeks in the case of castor oil plants: Sánchez-García et al., 2010). Thus, the expression of the genes of interest will have to be maintained after injection to induce changes in the oil composition of the endosperm, ruling out studies on dissected tissues and requiring protocols involving transient transformation *in planta*.

The present study is the first of a series that evaluate the use of the castor oil plant as a platform to produce oils of industrial interest. This requires the permanent transformation of the castor oil plant with constructs expressing or repressing distinct proteins and enzymes, and under the control of a variety of promoters. In these studies, it is essential to define a method to rapidly assess the activity of these constructs. As such, a method for the *in planta* transient transformation of castor endosperm was successfully developed, involving the injection of *Agrobacterium* into castor oil fruits under specific conditions. Once injected, the *Agrobacterium* could efficiently transform cells in the developing castor oil seed endosperm, which remained transformed throughout the period of castor oil accumulation. As a result, changes were induced in the oil composition of the mature seeds, allowing the effects of permanent transformation of castor embryos to be evaluated, along with the mRNA and protein expression driven by the different promoters. Accordingly, we transformed castor oil plant endosperm with a β-ketoacyl-CoA synthase (KCS) from *Lesquerella fendleri*, the enzyme responsible for the synthesis of lesquerolic acid (14-hydroxyl-eicosadec-11-enoic acid, 20:1-OH) from ricinoleic acid (Moon et al., 2001). The expression systems and the changes found in the final oil composition of transformed seeds were evaluated in light of these results. In addition, the potential and perspectives of using this new method on castor plant biotechnology are discussed.

## Materials and Equipment

### Biological Materials

Seeds from the isogenic *Ricinus communis* variety IN15 used in this study were kindly provided by Dr Leonardo Velasco (IAS, CSIC, Córdoba, Spain). The AGL1 *Agrobacterium tumefaciens* strain was supplied by professors Diego López Alonso and Federico García Maroto (University of Almería, Spain).

### Reagents and Media

Tryptone, yeast extract and bacto agar for LB medium elaboration and MS medium were purchased to Panreac Apli-chem (Madrid, Spain). The antibiotics kannamycin, rifampicin, and carbenicillin were provided by Thermo-Fisher Scientific (Waltham, MA, USA). Chemical reagents sucrose, acetosyringone, 5-bromo-4-chloro-3-indolyl glucuronide, KH_2_PO_4_, Triton X-100, potassium ferrocyanide, potassium ferricyanide, dithiothreitol, EDTA, sodium lauryl sarcosine, 4-trifluoromethylumbelliferyl-β-D-glucuronic acid, Na_2_CO_3_, 4-methyl umbelliferone, BSA, ammonium sulphate, trisodium citrate, citric acid, chloroacetaldehyde 0.5 M, hexamethyldisilazane, trimethylchlorosilane, and trimethylamine were acquired from Sigma-Aldrich (St. Louis, MO, USA). Dimethoxypropane, sulfuric acid, and HPLC grade Glacial acetic acid, methanol, toluene, chloroform and acetonitrile were purchased to Merk (Darmstadt, Germany). Acyl-CoA ester standards for the HPLC method calibration were provided by Avanti Polar Lipids (Alabaster, AL, USA).

### Molecular Biology

The pCAMBIA 1305.1 vector (Marker gene Technologies Inc., Eugene, OR, USA) was used for plant transformation constructions. The soybean glycinin promoter (661 bp) was obtained from the pBin plasmid, which was kindly provided by Professor Ed Cahoon (University of Nebraska).

The CFX96TM Real-Time Detection System using SYBR Green SsoAdvancedTM SYBR^®^ Green Supermix (Bio-Rad, Hercules, CA, USA) was used for studies of gene expression by rtPCR.

Total RNA was isolated using Spectrum Plant Total RNA Kit (Sigma-Aldrich, St. Louis, MO, USA). cDNA was synthesized from 1 µg of total RNA using Ready-To-Go T-Primed First-Strand Kit (GE Healthcare, Chicago, IL, USA).

EcoRI, NcoI restriction endonucleases and Klenow enzyme were purchased from New England Biolabs (Ipswich, MA). The T4 DNA Ligase was supplied by Thermo Fisher Scientific (Waltham, MA, USA).

### Equipment

The fluorescence was quantified using a Fluoroskan Ascent (Thermo Fisher, Waltham, MA, USA) multifluorescence scanner at excitation/absorption wavelengths of 355/460 nm.

GLC analysis was performed in a Hewlett–Packard 6890 gas chromatograph (Palo Alto, CA, USA) endowed with a flame ionization detector, using a Supelco SP-2380 fused-silica capillary column (30 m length, 0.25 mm i.d., 0.20 µm film thickness; Supelco, Bellefonte, PA, USA). The carrier gas used was hydrogen at 28 cm s^-1^, the temperature of the detector and injector was 200 °C, the oven temperature was 170 °C and the split ratio was 1:50. Peaks were identified by comparing their retention times with those of the corresponding commercial standards.

For HPLC analysis of acyl-CoA derivatives a Waters 2695 separation module endowed with a XBridge C18 column (250 × 0.5 mm, 5 µm: Waters) and a Multi λ Fluorescence Detector 2475 (Waters, Milford, MA, USA) was used.

## Methods

### Objective

The main objective of the method described in the present work is the *in planta* transient transformation of developing castor endosperm to make possible a fast testing of constructs and promoters before permanent transformation of this species. The transient expression of the exogenous gen expressed in this system should be high enough and last the time necessary to alter the fatty acid composition of the oil accumulated by the seed. So, we paid special attention to the expression levels at different days after injection (DAI). The method was tested by expressing an exogenous gene from *Lesquerella fedleri*.

### Plant Growth

Castor oil seeds were dehulled, sterilized with 50% sodium hypochlorite and germinated on wet perlite at 30°C. The seedlings were transferred to pots containing fertirrigation and grown in chambers at 26°C, on a 16h light/8h dark photoperiod and with 50% relative humidity.

### Vector Construction

The pCAMBIA 1305.1 vector was used for transient transformations (**Supplementary Figure 1**). For the construction of the pCAMBIA 1305.1:pGlycinin::GUS vector the CaMV 35S (35S) promoter was removed using the *EcoR*I and *Nco*I restriction endonucleases and the plasmid’s ends were then blunted with Klenow enzyme prior to relegating the plasmid using T4 DNA Ligase. The final construct was obtained by cloning the soybean glycinin promoter (661 bp) from the pBin plasmid (kindly provided by Dr Cahoon), generated as an *EcoR*I/*Nco*I fragment using specific primer pairs, into the GUS control vector with the same pair of restriction enzymes (**Supplementary Table 1**). For the pCAMBIA 1305.1:pGlycinin::LfKCS3 construct the *Lf*KCS3 open reading frame was re-synthesized *(GenScript Corporation,)* and codon-optimized for expression in *R. communis*. The LfKCS3 construct (1492 bp) was excised from the pUC57 plasmid (**Supplementary Figure 2**) using *Eco*RI and *Xho*I, and inserted into pBinGlyRed3 using T4 DNA ligase (Invitrogen). The LfKCS3 construct was flanked by a 5′-proximal glycinin promoter and a 3′ proximal glycinin UTR region. The 5′ pBinGlyRed3::LfKCS3::UTR 3′ assembly from pBIN was amplified with specific primers (**Supplementary Table 1**) and directionally cloned into the *Sac*I*-Kpn*I sites of the pCAMBIA 1305.1 vector. All the plasmids were verified by DNA sequencing and isolated from transformed XL-Blue *Escherichia coli* cells using the ISOLATE II plasmid mini kit (Bioline, London, UK) as DNA ready for *Agrobacterium* transformation.

### Castor Seeds Agroinfiltration

The AGL1 *Agrobacterium tumefaciens* strain was made CaCl_2_-competent and transformed by heat shock for 5 min at 37°C with the appropriate pCAMBIA 1305.1 construction, growing the transformed cells in LB plates containing the appropriate antibiotics (kanamycin, rifampicin and carbenicillin all at 50 mg/L). The day before infiltration, colonies were grown overnight at 28°C with shaking (230 rpm) in liquid LB medium (5 ml) supplemented with the mentioned antibiotics. Aliquots (0.2 ml) of the cultures were transferred to 50 ml fresh medium in a 250 ml flask, grown to O.D. = 0.2. Then, cells were then collected by centrifugation at 5000×*g* for 10 min and transferred to 50 ml MS medium, 30 g/L sucrose, 100 µM acetosyringone for agroinfiltration. Castor oil seeds were infiltrated with this cell suspension at different developmental stages. Infiltration was carried out with a 0.33 mm needle and the volume infiltrated was 300 µl, penetrating the fruit to reach the seed endosperm (**Supplementary Figure 3**). In order to prevent seed damage, a second hole was made to release the pressure in the seed.

### Histochemical Gus Assay

Histochemical characterization was carried out by incubating seed endosperm in a solution containing 5-bromo-4-chloro-3-indolyl glucoronide, 100 mM KH_2_PO_4_ buffer [pH 7], 0.06% Triton X-100, 5 mM potassium ferrocyanide and 5 mM potassium ferricyanide. Vacuum was applied to the tissues briefly before the seeds were transferred to a 70% ethanol solution and incubated at 37°C overnight in the dark.

### Fluorometric Determination of GUS Activity

Total GUS activity was measured in transiently transformed endosperm by fluorometry (Jefferson et al., 1987), first homogenizing the fresh endosperm (50–100 mg) in microcentrifuge tubes with 0.5 ml of ice cooled grinding buffer (50 mM KH_2_PO_4_ [pH 7.0], 10 mM, 1 mM EDTA, 0.1% sodium lauryl sarcosine and 0.1% Triton-X100). The homogenates were centrifuged at 13,000×*g* for 20 min at 4°C and the supernatants were used to assay GUS. The GUS assay mix contained 0.6 mM 4-trifluoromethylumbelliferyl-β-D-glucuronic acid in 225 µl grinding buffer and 50 µl of the crude supernatant. The assay mixture was incubated for 1h at 37°C and then 25 µl aliquots of the assay mix were added to 225 µl of 0.2 M Na_2_CO_3_. The fluorescence was then measured in a Fluoroskan Ascent multifluorescence scanner at excitation/absorption wavelengths of 355/460 nm. The fluorescence emitted was used to calculate activity on a fresh weight basis, in accordance with a calibration curve generated with different concentrations of 4-methyl umbelliferone assayed in the same system.

### mRNA Preparation and cDNA Synthesis

Approximately 0.3 g of agroinfiltrated castor seeds were ground in liquid nitrogen using precooled mortar and pestle. Total RNA was isolated using Spectrum Plant Total RNA Kit. cDNA was synthesized from 1 µg of total RNA using Ready-To-Go T-Primed First-Strand Kit.

### Quantitative Real-Time Polymerase Chain Reaction

The cDNA samples were subjected to quantitative real-time polymerase chain reaction (QRT-PCR) with specific pair of primers for the heterologous gene coding for GUS, Q-PCR-GUS-F (AACGACGGCAAGTTCCTCAT) and Q-PCR-GUS-R (CCATCACATTGCTCGCTTCG) on a CFX96TM Real-Time Detection System (Bio-Rad) using SYBR Green. Polymerase activation and DNA denaturation were performed at 95 °C for 30 s before carrying out 40 cycles of 95 °C for 15 s and 60 °C for 30s in which the fluorescence was monitored. Livack method (Livak and Schmittgen 2001) was used to calculate the comparative expression of the samples. The castor bean actin gene was used as calibrator gene and was amplified with the following specific pair of primers: qRcActin2_For (CCAGGGAGGAGTATGGAGGT) and qRcActin_Rev (ACCACATCCACAGGAACCAT).

### Determination of Fatty Acid Composition

Fatty acid composition was determined by direct methylation of the agroinfiltrated tissue followed of silanization and analysis by gas liquid chromatography (GLC) of the resulting silanized fatty acid methyl esters. The castor tissue was ground in 2 ml of a methanol/toluene/dimethoxypropane/sulfuric acid (33/14/20/10, by vol.) solution and then was heated at 80 °C for 1 h. In that conditions all fatty acids were transmethylated to their corresponding fatty acid methyl esters, which were later extracted with 3 ml of hexane. Hexane extracts were transferred to clean tubes and the solvent was removed under nitrogen flow to dryness. The residues were silanized with 0.1 ml of a hexamethyldisilazane/trimethylchlorosilane/pyridine mixture (3/1/5, by vol.) for 10 min. at room temperature to convert hydroxyl fatty acid methyl esters in their corresponding trimethyl-silyl derivatives. Then heptane was added to the mix to a final volume of 1.8 ml and methyl esters were analyzed by GLC in a Hewlett–Packard 6890 gas chromatograph endowed with a flame ionization detector, using a Supelco SP-2380 fused-silica capillary column (30 m length, 0.25 mm i.d., 0.20 µm film thickness; Supelco, Bellefonte, PA, USA). The carrier gas used was hydrogen at 28 cm s^-1^, the temperature of the detector and injector was 200 °C, the oven temperature was 170 °C and the split ratio was 1:50. Peaks were identified by comparing their retention times with those of the corresponding commercial standards.

### Acyl-CoA Pool Analysis

Acyl-CoAs were extracted from fresh developing castor oil plant endosperm and derivatized to their acyl-etheno derivatives, as indicated elsewhere (Larson and Graham, 2001). Endosperm tissue (150–250 mg) was crushed in a microcentrifuge tube after the addition of 1 nmol of heptadecanoyl-CoA as an internal standard and 250 µl of freshly prepared extraction buffer: 2 ml 2-propanol, 2 ml KH_2_PO_4_ 50 mM [pH 7.2], 50 µl glacial acetic acid and 80 µl BSA (50 mg/ml). The tissue was crushed with a micropestle, and the lipids extracted for 3 times with 200 µl hexane saturated with 2-propanol and water. Then, 5 µl of saturated ammonium sulphate were added and the protein was precipitated by adding 600 µl methanol/chloroform 2:1 v/v. The tubes were vortexed and incubated at room temperature for 20 min, and then finally centrifuged at 13,000×*g* for 2 min. The aqueous supernatants were then transferred to fresh tubes and dried under nitrogen at 40°C. The dried residues were dissolved in 40 µl chloroacetaldehyde reagent (0.5 M chloroacetaldehyde solution diluted in 0.15 M trisodium citrate/citric acid (pH 4.0), 0.5% w/v SDS) and analyzed by HPLC.

Acyl-CoA etheno derivatives were analyzed in a Waters 2695 separation module with a XBridge C18 column (250 × 0.5 mm, 5 µm: Waters) and a Multi λ Fluorescence Detector 2475 (Waters), and using a modified version of the quaternary gradient system described previously (Larson and Graham, 2001). The solvents consisted of A: 1% acetic acid; B: 90% acetonitrile, 1% acetic acid; C: 0.25%, trimethylamine, 0.1% tetrahydrofuran; D: 90% acetonitrile. The flow rate was 0.75 ml/min and the analysis temperature 40°C. The gradient elution profile followed the steps: 0–7.0 min, A–B 90:10 to A–B 20:80; 7.0–7.1 min, A–B 20:80 B to A–C 20:80; 7.1– 9.0 min, A–C 20:80 to C–D 90:10; 9.0–34.0 min, C–D 90:10 to C– D 25:75; 34.0–35.0 min, C–D 25:75 to D 100; 35.0–39.0 min, D 100; 39.0–40.0 min, D 100 to A–B 90:10; 40.0–45.0 min, A–B 90:10. The detector was set with an excitation wavelength of 230 nm and a detection wavelength of 420 nm. The peaks were quantified attending to the internal standard signal and the data were processed using the Empower Login software.

The non-commercial acyl-CoA standards like ricinoleoyl-CoA were prepared by reaction of the N-hydroxysuccinimide ester of the fatty acids with freshly reduced coenzyme A, as reported previously (Al-Arif and Blecher, 1969).

### Statistical Analysis

To establish significant differences between treatments one-way ANOVA was used combined with Tukey *post hoc* analysis with a significance level of 0.05%. The calculations were performed using a statistical package program OriginPro 2019 (Origin Lab Corporation, Northampton, MA, USA).

## Results and Discussion

### Transformation of the Developing Castor Oil Seed Endosperm

Castor oil seeds are covered with a hard testa that protects the endosperm and embryo. The endosperm accumulates large amounts of proteins and oil, which will be later used to feed the developing embryo during germination. The development of castor seeds does not differ from that reported for other oil seeds. The first stage involves tissue differentiation and cell division and it is followed by a stage in which the storage oil and proteins are synthesized. During the final stage of seed drying, all the biosynthetic activity is turned off. In the castor oil plant, the first stage occurs from day 1 to 10 after pollination (DAP) and the second one from 20 to 50 DAP, which is subsequently followed by the final drying stage (Sánchez-García et al., 2010). Here, a transient transformation method based on the direct injection of *Agrobacterium* into the capsule in which the seed endosperm was developing is reported. The vector used to study the efficiency of transformation was the pCAMBIA 1305.1, which contains a GUS reporter gene under the control of the strong constitutive 35S promoter from cauliflower mosaic virus (35S: see **Supplementary Figure 1**). Similar methods have been applied successfully in other plant transformation protocols, efficiently producing both permanent and transient transformants (Orzaez et al., 2006; Estrada-Navarrete et al., 2006). The Agro AGL1 strain was used here due its versatility and virulence.

**Figure 1:**
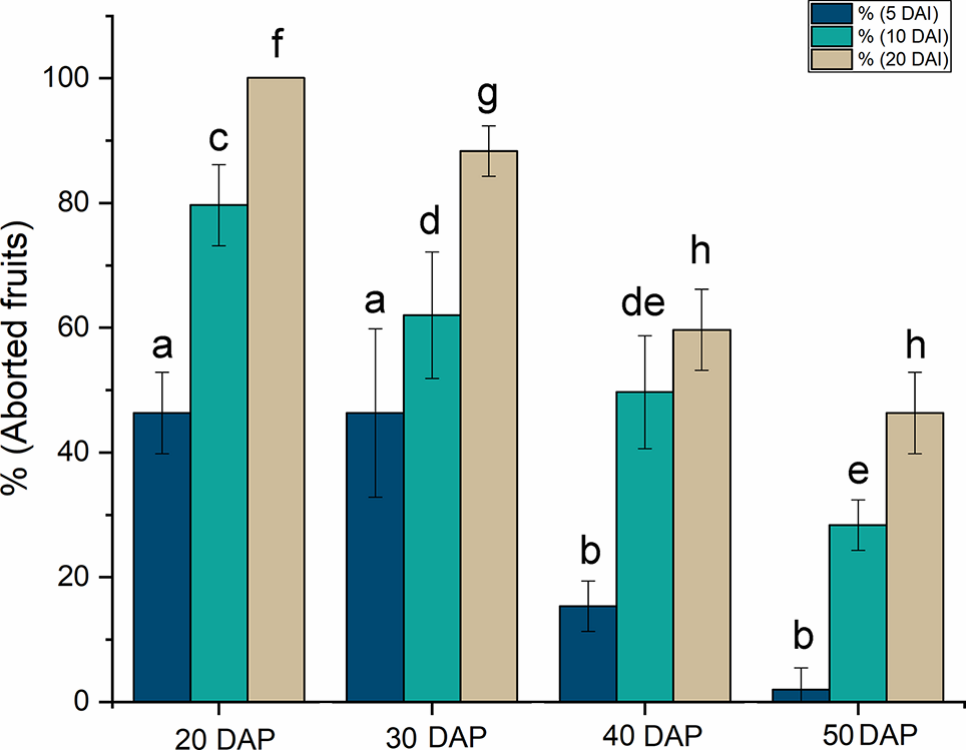
Effect of the stage of development on the rate of fruit abortion caused by the injection of *Agrobacterium*. The stage is expressed as days after pollination (DAP) and the abortion rates at 5, 10, and 20 days after injection (DAI) are shown. The data are the average of up to 10 injections ± the standard deviation. Letters indicate statistically significant differences (one-way ANOVA 0.05% significance level) when comparing aborted seeds at same DAI between seeds at different DAP.

Once the transformation protocol had been established, the influence of the developmental stage of the fruit on infiltration was investigated. This is a critical issue as injection at early stages of development means that the transformed cells can divide and propagate the expression of the target gene to a larger proportion of the cells in the mature seed. However, exposing the endosperm to *Agrobacterium* injection at such early stages can lead to seed abortion and lethality, reducing the survival of the transformed seeds. The process of castor development was depicted by Sánchez-García et al. (2010) and seven different stages of development were defined attending to morphological aspect. In this work we study the effect of agroinfiltration in seeds in stages 3, 4, 5 and 6, which approximately corresponded to 20, 30, 40, and 50 DAP. Seeds at 20 DAP have the endosperm tissue already differentiated and cells are still dividing. The oil accumulation starts around 30 DAP and reaches the highest rate at 40 DAP. This process slows down at 50 DAP, the point from which the seed drying stage takes place. Seeds were very susceptible to *Agrobacterium* inoculation at 20 DAP, when the protocol produced a large number of seed abortions, as high as 50% after 5 DAI, rising to 100% at 20 DAI, at the end of the studied period (**Figure 1**). Similar results were obtained with seeds injected at 30 DAP, with only slight differences in the rate of abortion at 20 DAI. At more advanced developmental stages, the rate of abortions decreased markedly at 5 DAI, reaching a value close to zero in mature seeds. At longer times (40 DAP), around 50–60% of the fruits inoculated aborted and this rate reached 30–50% when seeds were inoculated at 50 DAP (**Figure 1**). Since the objective of our studies was to investigate the influence of different genes on castor oil biosynthesis, these genes would need to be expressed during a sufficiently long period so as to affect oil composition while producing sufficient viable fruits. In view of the results obtained, the time chosen for fruit injection was 40 DAP, the moment in which high rate oil synthesis commences, the endosperm is well defined and the proportion of fruits that survived the injection was around 50% at 20 DAI.

**Figure 2:**
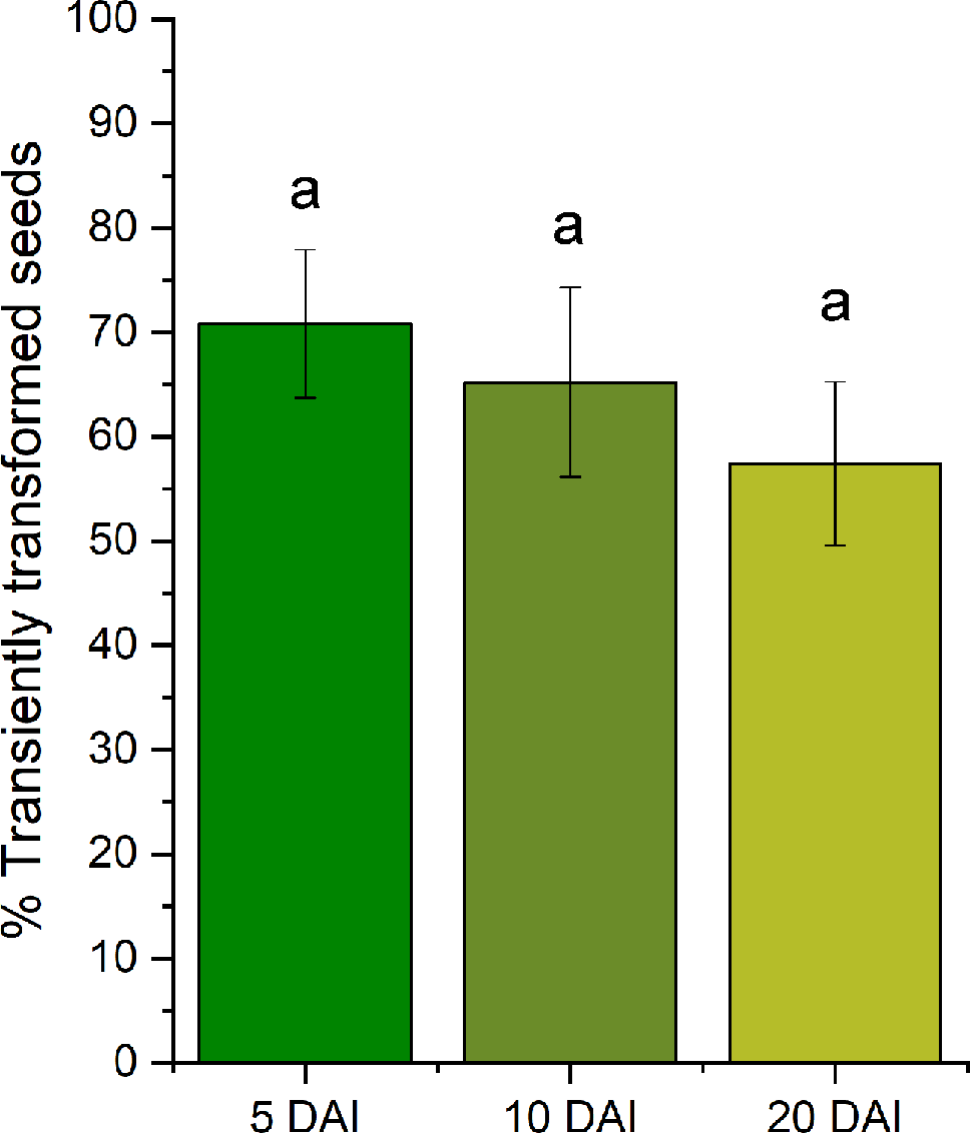
Survival rates of transformed seeds when they were injected at 40 days after pollination (DAP) and the data were obtained 5, 10, and 20 days after injection (DAI). Data represents the average of the survival rates of 4 groups of seeds injected the same day. The size of these groups were of 20 seeds for 5 DAI (20 × 4 = 80 total seeds), 18 seeds for 10 DAI (18 × 4 = 72 total seeds) and 19 seeds for 20 DAI (19 × 4 = 76 total seeds). Letters indicate statistically significant differences (one-way ANOVA 0.05% significance level).

**Figure 3:**
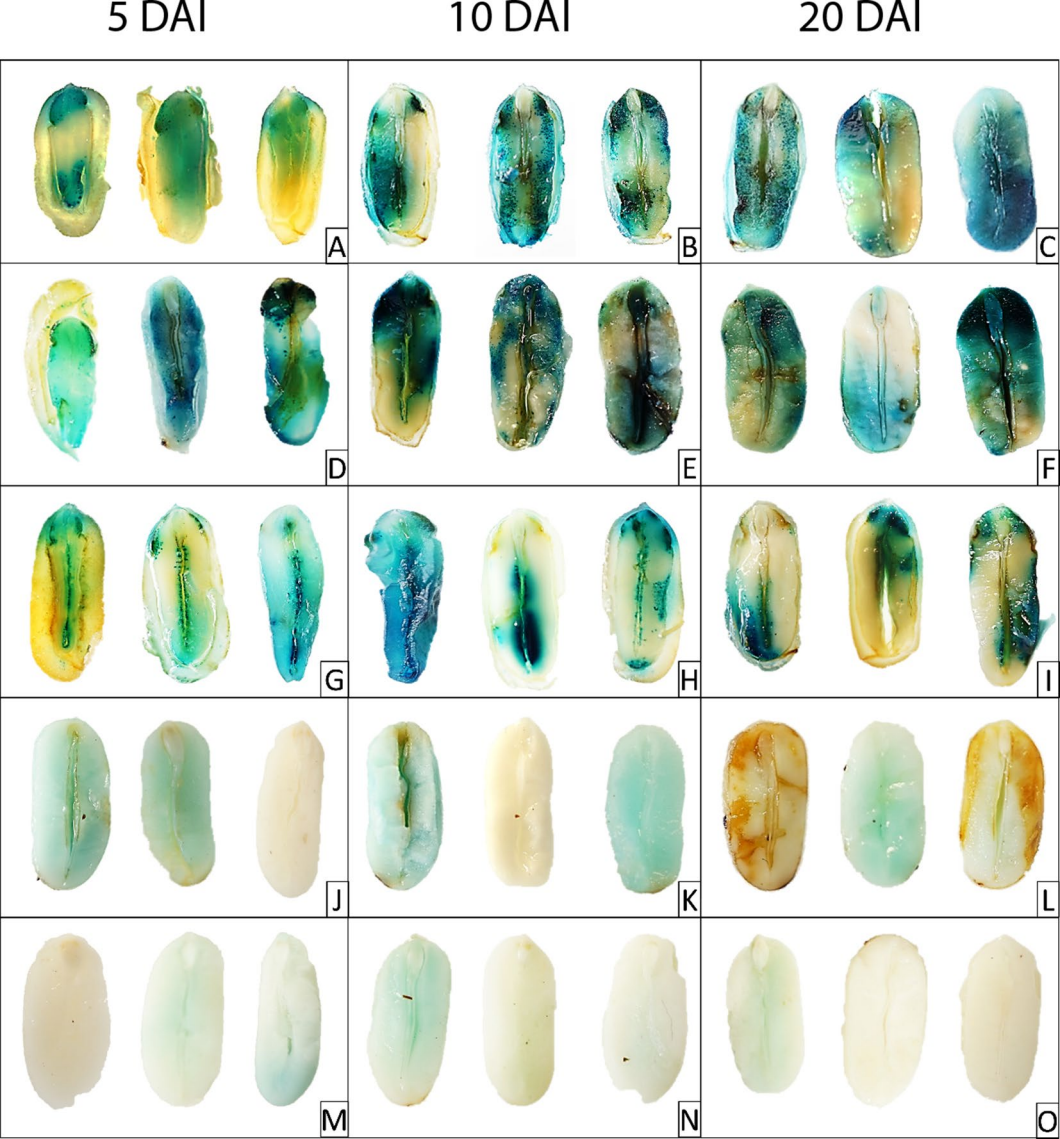
Galactouronidase (GUS) histochemical assay in castor endosperm transiently transformed using the protocol described herein. The three columns corresponded to endosperm injected with *Agrobacterium* and assessed 5, 10, or 20 days after injection (DAI). Each lane corresponds to the different constructs: **(A**–**C)**, were seeds transformed with the pCAMBIA 1305.1 vector; **(D**–**F)** correspond to pCAMBIA 1305.1 with GUS under the control of the glycinin promoter; **(G**–**I)** corresponded to the pCAMBIA 1305.1 containing glycinin::*Lf*KCS (plus 35S::GUS); **(J**–**L)** corresponded to the pCAMBIA 1305.1 vector without vector without a promoter; and **(M**–**O)**, were the control endosperm not injected with *Agrobacterium*.

Once these initial parameters had been established, the frequency of transformation was assessed in the surviving embryos to check if the T-DNA was truly transferred to the endosperm cells (**Figure 2**). GUS activity was measured in different groups of seeds infiltrated at 40 DAP and assayed at 5, 10, and 20 DAI, displaying transformation frequencies in the range between 60% and 70% of the seeds infiltrated. Given the reasonable proportion of viable transformants that completed all stages of seed development, it was possible to study processes occurring over longer periods and involving cell compartmentation, such as oil deposition. *Agrobacterium* was seen to transfer the T-DNA plasmid and the transformed endosperm cells displayed GUS activity. In the T-DNA plasmid used in the preliminary trials, GUS reporter gene expression was driven by the constitutive 35S promoter and its activity was evident in castor endosperm transiently transformed at different stages of development, as reflected by blue staining in the histochemical GUS assay (**Figure 3**). Indeed, the transformed castor endosperm developed a strong blue color at 5, 10, or 20 DAI when the reporter gene GUS was under the control of the constitutive 35S promoter present in pCAMBIA 1305.1 vector (**Figures 3A–C**). The blue staining was also very strong when that gene was regulated by the strong seed specific glycinin promoter (**Figures 3D–F**). Much less intense blue staining was seen when no promoter was included (**Figures 3J–L**). **Figures 3M–O** showed the blank of the method, corresponding to control experiments made with untransformed endosperm. The intensity of the color in the assay also varied from one seed to other depending on the proliferation of the *Agrobacterium* that took place in each individual case.

Unlike other systems, the expression of the exogenous genes remained for a long time. Thus, GUS activity was clearly detected 5 DAI and it remained active throughout the study, at 10 and 20 DAI (**Figure 3**). Dissected castor oil seed endosperm had previously been transformed *in vitro* (Chileh et al., 2010) but this system does not fully reproduce the physiological conditions of this process *in vivo*. In particular, the period of oil accumulation in castor plants lasts for around 40 days and thus, these preparations are not suitable to study the influence of expressing or repressing genes involved in this process. By contrast, transient transformation *in planta* opens new possibilities to study the synthesis of castor oil and proteins. In many transformed seeds the embryo was also transformed and thus, this new protocol for transient *in vivo* transformation could represent an efficient method to generate stable transgenic castor oil plants. This new protocol allowed us to rapidly perform proof of concept studies on the constructs, and to assay their impact on the process of oil and protein accumulation in this oil crop, potentially an important biotechnological platform. Moreover, since oil accumulation is regulated similarly in different plants, the platform presented here could represent a useful system to study metabolism in other developing oilseeds. Such studies could focus on testing constituent promoters in seeds, or on the overexpression or repression of different genes involved in lipid metabolism.

### The Testing of Different Promoters

**Figure 4:**
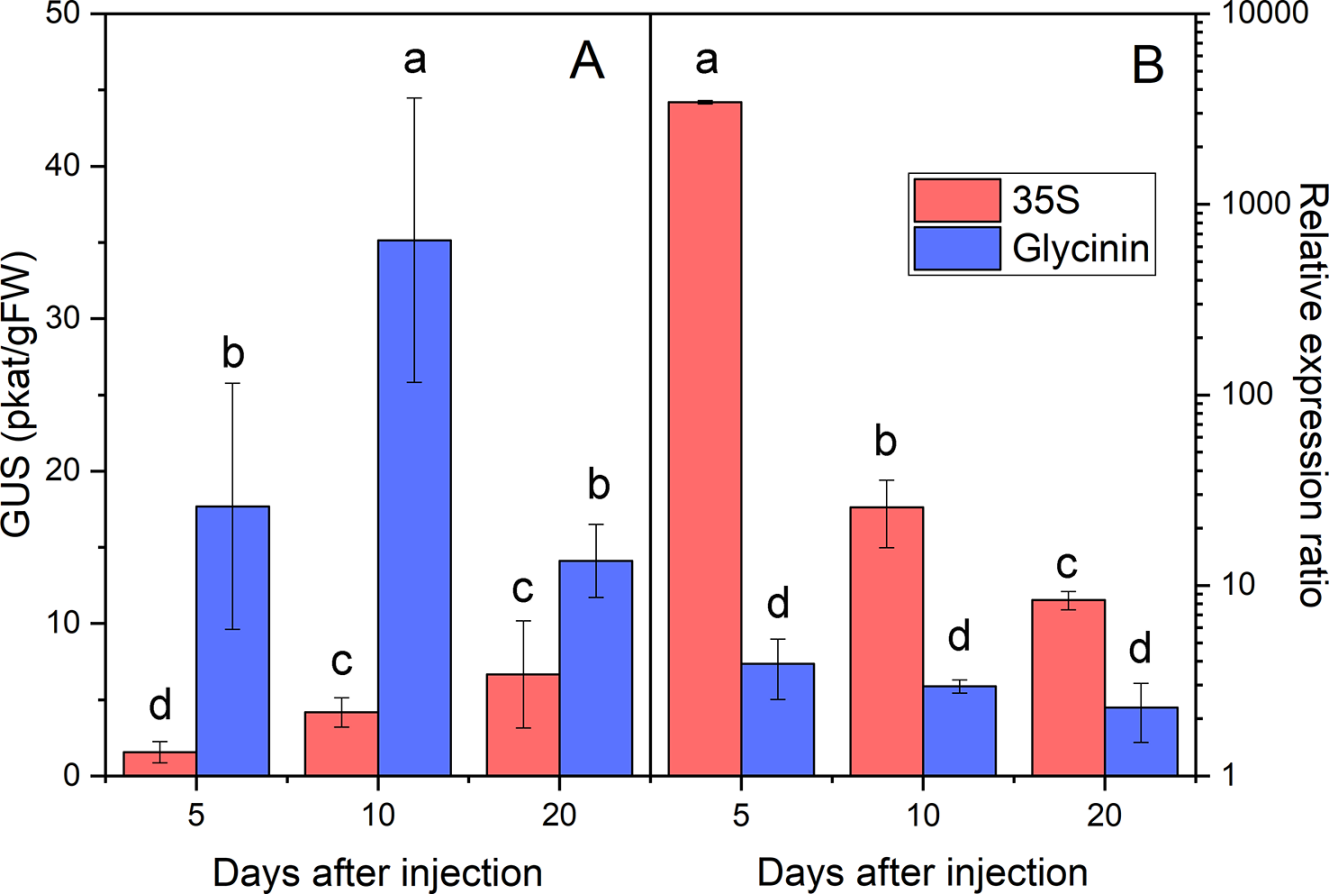
GUS marker activity measured by fluorometry **(A)** and number of GUS transcripts measured by QRT-PCR **(B)** in transiently transformed developing castor bean seed endosperm. The data corresponds to the GUS marker driven by the CaMV 35S (red) and glycinin (blue) promoter. Data are the average ± SD of five endosperms harvested at 5, 10, and 20 days after injection (DAI). Letters, independents for each panel, indicate statistically significant differences (one-way ANOVA 0.05% significance level).

To initially assess the suitability of the system developed to study the physiology of oil accumulation in castor oil endosperm, we quantified the expression of the marker gene from the moment of transformation up to 20 DAI, both in terms of transcription and translation. Preliminary trials were run using the GUS gene under the control of the strong, constitutive 35S promoter expressed in castor oil seeds at 40 DAP, at the beginning of seed filling. Infection of *Agrobacterium* took place over very short times and reporter gene expression was detected a few hours after injection (data not shown), with GUS gene expression increasing significantly in the following days. When driven by the 35S promoter, large numbers of GUS transcripts were found at 5 DAI, which later decreased at 10 and 20 DAI (**Figure 4B**). The level of GUS activity displayed less variation and tends to increase slightly over time, reaching a maximum at 10-20 DAI (**Figure 4A**).

Most previously described transient transformation procedures use the 35S promoter (Wroblewski et al., 2005; Carvalho et al., 2016), as it is a constitutive promoter that produces large numbers of transcripts in all tissues. Nevertheless, this promoter is not often used in seed biotechnology because such strong overexpression of the target gene/protein could affect vegetative tissue, harming plant growth and development. Moreover, there are many seed specific promoters that can also produce large numbers of transcripts. Therefore, transient transformation trials were carried out using the strong seed specific soybean glycinin promoter (Nagasawa and Oeda, 1995), which has been used extensively to express genes in this organ (Zhang et al., 2018). In these trials, the number of transcripts and the activity of the GUS marker were determined up to 20 DAI. While the maximal transcript expression driven by 35S was reached at 5 DAI, decreasing thereafter, the glycinin promoter drove the accumulation of fewer transcripts but at a more stable level of expression, remaining relatively constant at the three time points studied (**Figure 4**). Indeed, while the relative expression achieved with the 35S promoter was always one to three orders of magnitude higher in the transformed endosperm than that obtained with the glycinin promoter, GUS activity under the control of the glycinin promoter was always higher than that driven by the 35S promoter (**Figure 4**), reaching a maximum at 10 DAI albeit decreasing markedly at 20 DAI. This higher activity was also evident in the histochemical assay (**Figure 3**), in which endosperm transformed with glycinin-regulated vector (**Figures 3**) had a more intense color than that produced by the original vector with the 35S promoter (**Figures 3**). Hence, the strong expression of transgenes under the control of the 35S promoter is associated with a lower rate of translation of the transcripts in the developing castor oil seed endosperm. This reduction in translation was not so evident with the glycinin promoter, which produces more GUS activity over the time course of the experiment. Hence, there appear to be mechanisms that inhibit the translation of strongly expressed genes in castor oil seeds.

Silencing mechanisms have been observed in plant biotechnology from its very outset, and it is often triggered when genes are expressed under the control of strong promoters like 35S (Elmayan and Vaucheret, 1996), involving epigenetic mechanisms and reflecting a genome surveillance system that eliminates the RNA of excessively transcribed genes (Schubert et al., 2004). Those silencing events have been also reported in transient expression as a response of the plant to viral infections (Angell and Baulcombe, 1997) In this regard, Johansen and Carrington (2001) studied this process in transiently transformed tobacco leaves, depicting a complex regulation mechanism that depends on factors able of inhibiting or trigger gene silencing (Johansen and Carrington, 2001). Gene silencing produces a decrease in both the number of transcripts and the amount of translated protein. Here, we observed decreases in the activity of the reporter gene while the transcript levels remained high, suggesting that mechanisms other than transcript degradation were at play. Nevertheless, promoters like glycinin that induce more specific and moderate transcription rate seem to be more appropriate for transient transformation, particularly when used to gain insight into the events controlling oil biosynthesis in castor bean plants. A similar result was reported by Orzaez et al. (2006) in tomato fruit, where low levels of GUS and yellow fluorescence protein markers were detected in tomato fruit when they were transiently expressed under the control of 35S. This problem was overcome by using an Arabidopsis heat shock promoter, which allowed GUS activity to be detected only when the fruit was maintained at 45°C. Furthermore, studies of transient expression in other oil crops like soybean did not detected any silencing induced by 35S in that seed endosperm (King et al., 2015), so such events may be very dependent on the plant host species. Nevertheless, the data presented here do indicate that castor oil plants can trigger this defense mechanism, which must be taken into consideration when performing transformations of this plant. This could involve using promoters with moderate levels of expression or including silencing suppressors like P19 or V2 within the transformation vectors. Therefore, the following experiment was carried out using the seed specific promoter glycinin, which produces reasonable high levels of protein translation in transiently transformed endosperm.

### Transient Expression of *Lesquerella fendleri* KCS in Castor Endosperm

**Figure 5:**
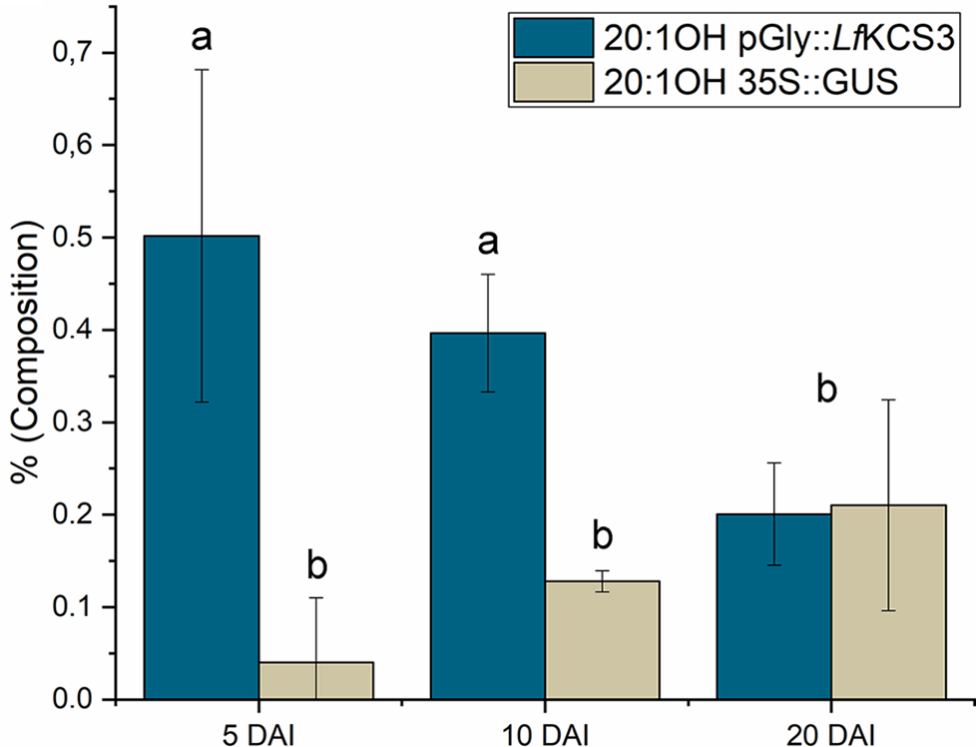
Variation in the castor endosperm lesquerolic acid content induced by transient expression of *L. fendleri* β-ketoacyl-CoA synthase. The data correspond to the endosperm harvested 5, 10, and 20 days after injection (DAI), and they are the average of up to 4-6 injections ± the standard deviation. The data are the average ± the standard deviation corresponding to 4 endosperms harvested at 5, 10, and 20 days after injection (DAI). Letters indicate statistically significant differences (one-way ANOVA 0.05% significance level).

Once the transformation protocol was optimized, the next objective was to test if it was possible to alter the castor oil composition by expressing constructions designed for it. In this regard, the *L. fendleri* species contains high levels of lesquerolic acid ((11Z, 14R)-14-hydroxyicos-11-enoic acid, 20:1-OH), an analogue of ricinoleic acid generated by elongation through the action of a desaturase/elongase and a specific KCS (Moon et al., 2001). Since castor oil plants produce the ricinoleic acid precursor, they were transformed with a construct expressing the *L. fendleri* KCS (*Lf*KCS) under the control of glycinin promoter. This *LfKCS* gene was codon-optimized for stronger expression in castor oil seeds by GenScript^®^, and the fatty acid composition of the transformed endosperm was assessed 5, 10, and 20 DAI, as well as the *LfKCS* transcripts. The percentage of embryos being transformed after infiltration with this construct did not differ much from results in **Figure 2** (data not shown). This construct also expressed the GUS marker under the control of 35S, which was used to confirm that the tissue was correctly transformed using the histological GUS assay (**Figures 3**). Furthermore, PCR analysis confirmed the presence of *LfKCS* and the *GUS* marker transcripts in the transiently transformed endosperm (**Supplementary Figure 4**), and when the fatty acid composition of the endosperm was assessed, there was a significant increase in very long chain fatty acids at 5 and 10 DAI (**Figure 5**).

Castor oil has small amounts of lesquerolic acid, indicating that there is some endogenous elongase activity in their endosperm cells. However, at 5 DAI there were important changes in lesquerolic acid in the transiently transformed seeds, which increased 8-fold. The increase of lesquerolic was also significant at 10 DAI, where it was threefold higher than in the control endosperm transformed with the empty pCAMBIA 1305.1 plasmid. These differences were not observed after 20 DAI, when there was an equivalent amount of lesquerolic in the transformed and control endosperm. The expression of the *LfKCS* gene followed the pattern described above for genes regulated by glycinin (**Figure 4**).The effect on transgene expression was largely reversed when most of the oil was accumulated in the castor oil seeds. Nevertheless, although there was an important increment of lesquerolic acid at 5 and 10 DAI in this experiment, this fatty acid did not exceed 0.5% of the total fatty acids. Hence, the stable expression of this gene in castor endosperm probably would not produce excessive accumulation of this fatty acid.

**Figure 6:**
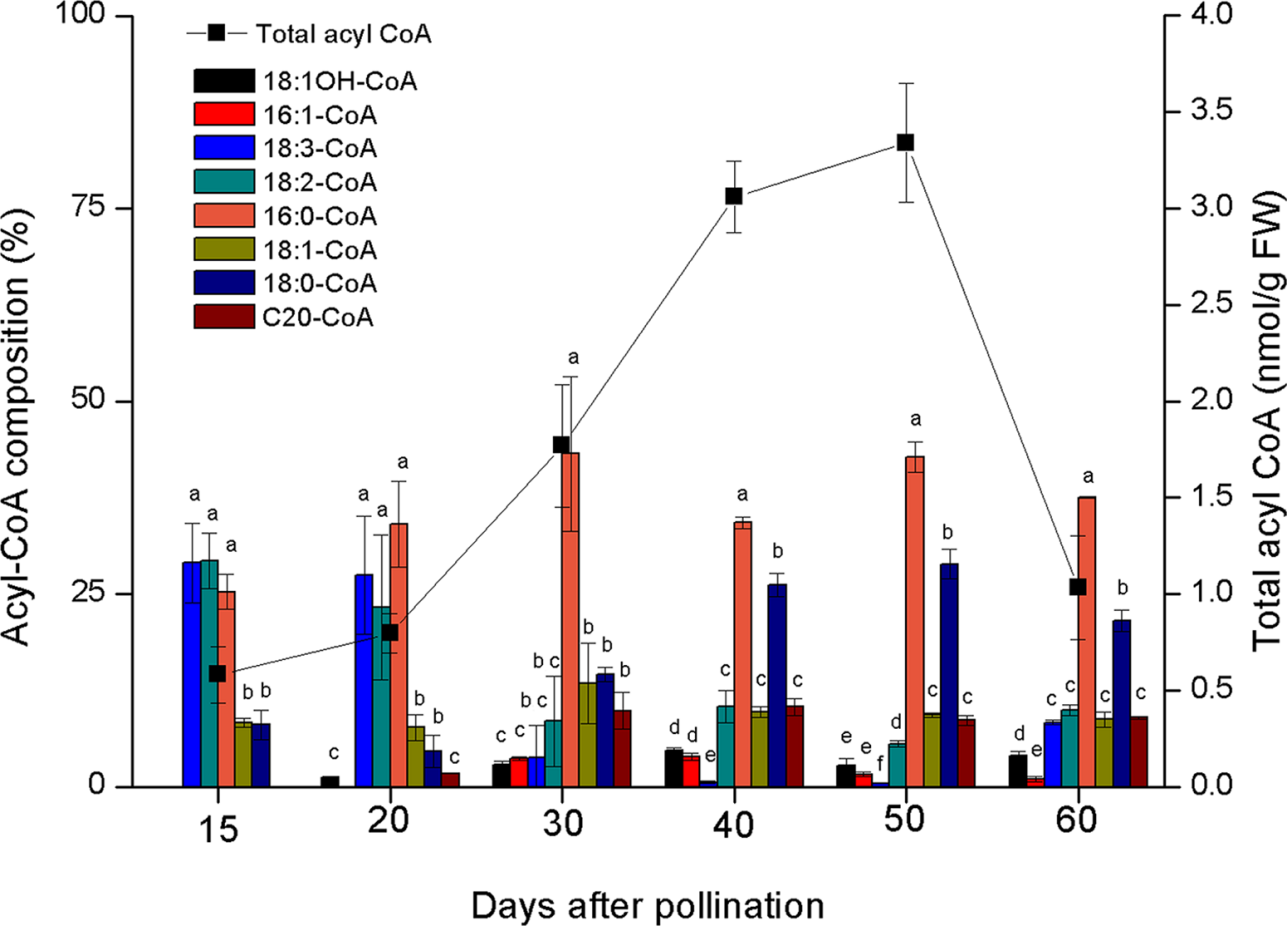
Composition and size of the castor acyl-CoA pool during seed development. The data are the average of three to four independent determinations. The data are the average ± SD of 4 independent determinations. Letters indicate statistically significant differences (one-way ANOVA 0.05% significance level) when comparing acyl-CoAs at same days after anthesis samples.

Lesquerolic acid is synthesized through the elongation of ricinoleoyl-CoA and castor oil is very rich in ricinoleic acid, although the synthesis of this oil involves the quick and efficient channelling of this fatty acid into TAGs in oil bodies. This channelling could involve the efficient removal of ricinoleoyl-CoA from the acyl-CoA pool by reticular acyltransferases, which would deplete the cytosol of the physiological substrate of *Lf*KCS. This hypothesis was investigated by examining the composition of the acyl-CoA pool in the developing castor oil seed endosperm (**Figure 6**). Among the acyl-CoA species present in the cytosol of endosperm cells in the castor oil seed, ricinoleoyl-CoA is underrepresented relative to other acyl species, like palmitoyl-, oleoyl-, and linoleoyl-CoAs, suggesting that the weak production of lesquerolic acid by *Lf*KCS may be related to poor substrate availability. In this regard, when the incorporation of different fatty acids into castor microsomes was studied, ricinoleic acid was the most prolific, followed by linoleic and oleic acid, and then saturated species (Lin et al., 2002). The weaker incorporation of those fatty acids was consistent with their stronger contribution to the acyl-CoA pool of this species. Furthermore, we have to consider that the elongase activity is not due to a single gene (the condensing enzyme or KCS), but it is a type II complex comprising other activities like ketoacyl-CoA dehydrogenases, hydroxyacyl-CoA dehydrases, or enoyl-CoA reductases. These enzymes are not often considered limiting, and we have assumed they are present within the enzymatic dotation of castor at a level enough to complete the elongation of ricinoleic acid. A deficiency in the activity of any of those enzymes in castor endosperm could also be responsible of a low production of lesquerolic acid.

## Conclusions

A method to transiently transform castor oil plant endosperm is presented in this work, based on by direct *Agrobacterium* injection into castor fruit. This method was consistent and reproducible, producing large proportions of viable transformed seeds when injections were performed up to 40 DAP, and transgene expression for longer than 15 days. While the constitutive 35S promoter drove very high levels of expression, even at short times after injection, the seed specific promoter glycinin produced fewer transcripts but stronger activity of the GUS marker. Hence, a defense mechanism may be triggered when the transgene was expressed under the constitutive 35S promoter. When the impact of expressing *LfKCS* in developing castor seeds was investigated, the expression of this gene caused a significant increase in the elongation of ricinoleic to lesquerolic acid in transiently transformed endosperm. However, large amounts of this product did not accumulate, possibly due to the poor availability of ricinoleoyl-CoA in the castor acyl-CoA pool.

## Author Contributions

AS-Á performed most of the experimental work. NR-L participated in plasmid construction and experimental design. AM-P performed the rtPCR determinations. JS performed the GUS activity determination. EM-F, RG, and JS participated in the work direction and experimental design. JS and NR-L wrote and revised the manuscript.

## Funding

This work was funded by the Junta de Andalucía project P12-AGR-543, Spain (2012), and AS- Á was supported by a Junta de Andalucía predoctoral fellowship.

## Conflict of Interest

The authors declare that the research was conducted in the absence of any commercial or financial relationships that could be construed as a potential conflict of interest.

## Abbreviations

DAI, days after imbibition; TAG, triacylglycerol; PDCT, PC-diacylglycerol phosphocholine transferase; KCS, β-ketoacyl-CoA synthase; DAP, days after pollination; GUS, galactouronidase; 35S, CaMV 35S cauliflower mosaic virus; RNA, ribonucleic acid; DNA, deoxyribonucleic acid; *Lf*KCS, *Lesquerella fendleri* β-ketoacyl-CoA synthase.

## References

[B1] Ahn Y. J. Vang L. McKeon T. A. Chen G. Q. (2007). High-frequency plant regeneration through adventitious shoot formation in castor (*Ricinus communis* L.). In Vitro Cell. Dev. Biol. Plant 43, 9–15. 10.1007/s11627-006-9009-2

[B2] Al-Arif A. Blecher M. (1969). Synthesis of fatty acyl-CoA and other thiol esters using N-hydroxysuccinimide esters of fatty acids. J. Lipid Res. 10, 344–345. ISSN 0022-2275. 5785007

[B3] Angell S. M. Baulcombe D. C. (1997). Consistent gene silencing in transgenic plants expressing a replicating potato virus X RNA. EMBO J. 16, 3675–3684. 10.1093/emboj/16.12.3675 9218808 PMC1169991

[B4] Bafor M. Smith M. A. Jonsson L. Stobart K. Stymne S. (1991). Ricinoleic acid biosynthesis and triacylglycerol assembly in microsomal preparations from developing castor-bean (*Ricinus communis*) endosperm. Biochem. J. 280, 507–514. 10.1042/bj2800507 1747126 PMC1130577

[B5] Baldwin B. S. Cossar R. D. (2009). Castor yield in response to planting date at four locations in the south-central United States. Ind. Crops Prod. 29, 316–319. 10.1016/j.indcrop.2008.06.004

[B6] Berman P. Nizri S. Wiesman Z. (2011). Castor oil biodiesel and its blends as alternative fuel. Biomass Bioenergy 35, 2861–2866. 10.1016/j.biombioe.2011.03.024

[B7] Carvalho R. F. Carvalho S. D. O’Grady K. Folta K. M. (2016). Agroinfiltration of strawberry fruit - A powerful transient expression system for gene validation. Curr. Plant Biol. 6, 19–37. 10.1016/j.cpb.2016.09.002

[B8] Chan A. P. Crabtree J. Zhao Q. Lorenzi H. Orvis J. Puiu D. (2010). Draft genome sequence of the oilseed species *Ricinus communis* . Nat. Biotechnol. 28, 951–956. 10.1038/nbt.1674 20729833 PMC2945230

[B9] Chileh T. Esteban-García B. Alonso D. L. García-Maroto F. (2010). Characterization of the 11S globulin gene family in the castor plant *Ricinus communis* L. J. Agric. Food Chem. 58, 272–281. 10.1021/jf902970p 19908832

[B10] Das G. Trivedi R. K. Vasishtha A. K. (1989). Heptaldehyde and undecylenic acid from castor oil. J. Am. Oil Chem. Soc. 66, 938–941. 10.1007/BF02682613

[B11] Dwivedi M. C. Sapre S. (2002). Total vegetable-oil based greases prepared from castor oil. Lubrication Sci. 19, 229–241. 10.1002/jsl.3000190305

[B12] Elmayan T. Vaucheret H. (1996). Expression of single copies of a strongly expressed 35S transgene can be silenced post-transcriptionally. Plant J. 9, 787–797. 10.1046/j.1365-313X.1996.9060787.x

[B13] Estrada-Navarrete G. Alvarado-Affantranger X. Olivares J. E. Díaz-Camino C. Santana O. Murillo E. (2006). *Agrobacterium rhizogenes* transformation of the *Phaseolus* spp.: a tool for functional genomics. Mol. Plant-Microbe Interact. 19, 1385–1393. 10.1094/MPMI-19-1385 17153923

[B14] Han L. Zhang L. Liu J. Li H. Wang Y. Hasi A. (2015). Transient expression of optimized and synthesized nattokinase gene in melon (*Cucumis melo* L.) fruit by agroinfiltration. Plant Biotechnol. 32, 175–180. 10.5511/plantbiotechnology.15.0430a

[B15] Hu Z. Ren Z. Lu C. (2012). The phosphatidylcholine diacylglycerol choline phosphotransferase is required for efficient hydroxy fatty acid accumulation in transgenic Arabidopsis. Plant Physiol. 158, 1944–1954. 10.1104/pp.111.192153 22371508 PMC3320197

[B16] Jefferson R. A. Kavanagh T. A. Bevan M. W. (1987). GUS fusions betaglucuronidase as a sensitive and versatile gene fusion marker in higher plants. EMBO J. 6, 3901–3907. 10.1002/j.1460-2075.1987.tb02730.x 3327686 PMC553867

[B17] Johansen L. K. Carrington J. C. (2001). Silencing on the spot. Induction and suppression of RNA silencing in the *Agrobacterium*-mediated transient expression system. Plant Physiol. 126, 930–938. 10.1104/pp.126.3.930 11457942 PMC1540124

[B18] King J. L. Finer J. J. McHale L. K. (2015). Development and optimization of agroinfiltration for soybean. Plant Cell Rep. 34, 133–140. 10.1007/s00299-014-1694-4 25326714

[B19] Kroon J. T. Wei W. Simon W. J. Slabas A. R. (2006). Identification and functional expression of a type 2 acyl-CoA: diacylglycerol acyltransferase (DGAT2) in developing castor bean seeds which has high homology to the major triglyceride biosynthetic enzyme of fungi and animals. Phytochem. 67, 2541–2549. 10.1016/j.phytochem.2006.09.020 17084870

[B20] Larson T. R. Graham I. A. (2001). Technical advance: a novel technique for the sensitive quantification of acyl CoA esters from plant tissues. Plant J. 25, 115–125. 10.1111/j.1365-313X.2001.00929.x 11169187

[B21] Lin J. T. Chen J. M. Liao L. P. McKeon T. A. (2002). Molecular species of acylglycerols incorporating radiolabeled fatty acids from castor (*Ricinus communis* L.) microsomal incubations. J. Agric. Food Chem. 50, 5077–5081. 10.1021/jf020454a 12188611

[B22] Livak K. J. Schmittgen T. D. (2001). Analysis of relative gene expression data using real-time quantitative PCR and the 2(-Delta Delta C(T)) method. Methods 25, 402–408. 10.1006/meth.2001.1262 11846609

[B23] Moon H. Smith M. A. Kunst L. (2001). A condensing enzyme from the seeds of *Lesquerella fendleri* that specifically elongates hydroxy fatty acids. Plant Physiol. 127, 1635–1643. 10.1104/pp.010544 11743108 PMC133568

[B24] Mutlu H. Meier M. A. (2010). Castor oil as a renewable resource for the chemical industry. Eur. J. Lipid Sci. Technol. 112, 10–30. 10.1002/ejlt.200900138

[B25] Nagasawa A. Oeda K. (1995). Positive and negative cis-regulatory regions in the soybean glycinin promoter identified by quantitative transient gene expression. Plant Cell Rep. 14, 539–544. 10.1007/BF00231934 24185593

[B26] Orzaez D. Mirabel S. Wieland W. H. Granell A. (2006). Agroinjection of tomato fruits. A tool for rapid functional analysis of transgenes directly in fruit. Plant Physiol. 140, 3–11. 10.1104/pp.105.068221 16403736 PMC1326026

[B27] Potrykus I. (1991). Gene transfer to plants: assessment of published approaches and results. Ann. Rev. Plant Biol. Plant Mol. Biol. 42, 205–225. 1040-2519/9110601-0205

[B28] Sánchez-García A. Moreno-Pérez A. J. Muro-Pastor A. M. Salas J. J. Garcés R. Martínez-Force E. (2010). Acyl-ACP thioesterases from castor (*Ricinus communis* L.): an enzymatic system appropriate for high rates of oil synthesis and accumulation. Phytochem. 71, 860–869. 10.1016/j.phytochem.2010.03.015 20382402

[B29] Schubert D. Lechtenberg B. Forsbach A. Gils M. Bahadur S. Schmidt R. (2004). Silencing in Arabidopsis T-DNA transformants: the predominant role of a gene-specific RNA sensing mechanism versus position effects. Plant Cell 16, 2561–2572. 10.1105/tpc.104.024547 15367719 PMC520955

[B30] Sousa N. L. Cabral G. B. Vieira P. M. Baldoni A. B. Aragão F. J. (2017). Bio-detoxification of ricin in castor bean (*Ricinus communis* L.) seeds. Sci. Rep. 7, 15385–15363. 10.1038/s41598-017-15636-7 PMC5684206 29133924

[B31] Sujatha M. Sailaja M. (2005). Stable genetic transformation of castor (*Ricinus communis* L.) *via Agrobacterium tumefaciens*-mediated gene transfer using embryo axes from mature seeds. Plant Cell Rep. 23, 803–810. 10.1007/s00299-004-0898-4 15580353

[B32] Thakur S. Karak N. (2013). Castor oil-based hyperbranched polyurethanes as advanced surface coating materials. Prog. Org. Coatings. 76, 157–164. 10.1016/j.porgcoat.2012.09.001

[B33] Van De Loo F. J. Broun P. Turner S. Somerville C. (1995). An oleate 12-hydroxylase from *Ricinus communis* L. is a fatty acyl desaturase homolog. Proc. Nat. Acad. Sci. U.S.A. 92, 6743–6747. 10.1073/pnas.92.15.6743 PMC41405 7624314

[B34] Van Erp H. Bates P. D. Burgal J. Shockey J. (2011). Castor phospholipid: diacylglycerol acyltransferase facilitates efficient metabolism of hydroxy fatty acids in transgenic Arabidopsis. Plant Physiol. 155, 683–693. 10.1104/pp.110.167239 21173026 PMC3032459

[B35] Venegas-Calerón M. Sánchez R. Salas J. J. Garcés R. Martínez-Force E. (2016). Molecular and biochemical characterization of the OLE-1 high-oleic castor seed (*Ricinus communis* L.) mutant. Planta 244, 245–258. 10.1007/s00425-016-2508-4 27056057

[B36] Wroblewski T. Tomczak A. Michelmore R. (2005). Optimization of *Agrobacterium*-mediated transient assays of gene expression in lettuce, tomato and Arabidopsis. Plant Biotechnol. J. 3, 259–273. 10.1111/j.1467-7652.2005.00123.x 17173625

[B37] Zhang N. McHale L. K. Finer J. J. (2018). Changes to the core and flanking sequences of G-box elements lead to increases and decreases in gene expression in both native and synthetic soybean promoters. Plant Biotechnol. J. 17, 724–735. 10.1111/pbi.13010 30191675 PMC6419578

