# Supplementary material for: Agrobacterium-Mediated Transient Gene Expression in Developing Ricinus communis Seeds: A First Step in Making the Castor Oil Plant a Chemical Biofactory
Source: Front Plant Sci. 2019 Oct 30;10:1410. doi: 10.3389/fpls.2019.01410 (PMC6831639; doi:10.3389/fpls.2019.01410)
Supplement: Supplementary file 1 [file DataSheet_1.pdf]

Supplementary table 1

Oligo name	Orientation	Sequence (5'-3')	PCR fragment size
pGly_prom_SacI	Forward	AG**GAGCTC**GTACGTAAGTACGTACT	2669bp
pGly_UTR_KpnI	Reverse	AGA**GGTACC**TAAGTCATGAAGAACCTG	
pGly_Eco	Forward	CTG**GAATTT**GTACGTACTCAAAAT GCCAA	661bp
pGly_Nco	Reverse	ATC**CCATGG**TTAAGGACCAATGGAGAGAA	
*Lf*KCS3_Fw	Forward	CATTAAGAGTTTCAATCTTGGTGGAATGG	338bp
*Lf*KCS3_Rv	Reverse	GGAAGAGGATGAAATGGGTAAAGTTGGAG	
GusPlus_Fw	Forward	CCGCACACTATCCGTACTC	161bp
GusPlus_Rv	Reverse	GTACCTGGGAGAAGATTCGG	

Figure 1

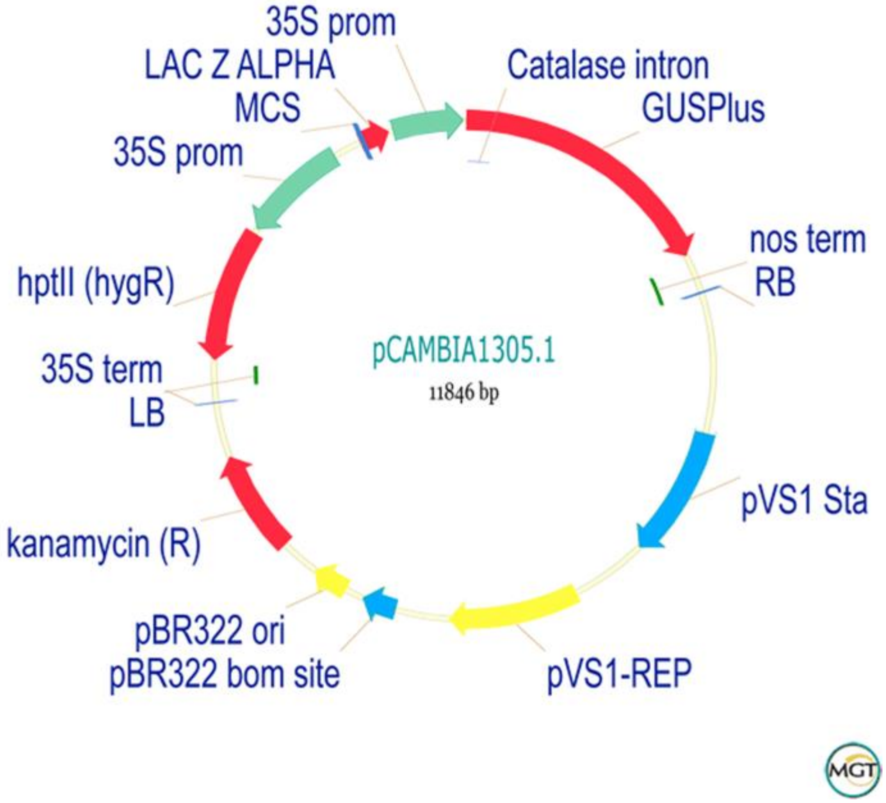

Map of pCAMBIA 1305.1 vector

Figure 2

Sequences of *Lesquerella fedleri* KCS, original and codon-optimized (modified).

```
                           10        20        30        40        50        60
                   ....|....|....|....|....|....|....|....|....|....|....|....|
LfKCS3 original    ATGACATCTCTAAACATAAAGCTCCTTTACCATTACATCCTAACCAACCTTTTCAACCTC
LfKCS3 modified    ATGACCTCTCTTAACATCAAACTTCTTTATCACTACATCCTTACTAATCTTTTCAACTTG
                   ***** ***** ***** ** ** ***** ** ******** ** ** ********* * 

                           70        80        90       100       110       120
                   ....|....|....|....|....|....|....|....|....|....|....|....|
LfKCS3 original    TGTCTCTTCCCTTTAACAGCATTTCTCGCCGGAAAAGCTTCTCACTTAACCAAATCCGAT
LfKCS3 modified    TGTCTTTTCCCACTTACTGCTTTTCTTGCTGGTAAAGCAAGTCATCTTACTAAGTCTGAT
                   ***** *****  * ** ** ***** ** ** *****   ***  * ** ** ** ***

                          130       140       150       160       170       180
                   ....|....|....|....|....|....|....|....|....|....|....|....|
LfKCS3 original    CTCCTCATGTTCTTATCTCATCTCCAAGACAATCTTATAACTGTCATTGTACTCTTTACT
LfKCS3 modified    CTTTTGATGTTCTTGTCTCACCTTCAAGATAATTTGATCACAGTTATAGTGCTTTTTACC
                   **  * ******** ***** ** ***** *** * ** ** ** ** ** ** ***** 

                          190       200       210       220       230       240
                   ....|....|....|....|....|....|....|....|....|....|....|....|
LfKCS3 original    TTCACTATCTTCTGTTTGGTTCTTTACATTGTAACCAAACCTAAACAGATTTATCTTGTG
LfKCS3 modified    TTCACTATCTTCTGCCTTGTTTTGTATATTGTGACTAAGCCAAAGCAGATATACCTTGTT
                   **************  * *** * ** ***** ** ** ** ** ***** ** ***** 

                          250       260       270       280       290       300
                   ....|....|....|....|....|....|....|....|....|....|....|....|
LfKCS3 original    GATTACTCTTGTTACCTTCCACCAGATCATCTTAAAGTTAGTATATCAAGTGTCATGGAT
LfKCS3 modified    GATTACTCATGTTACTTGCCACCTGATCATCTTAAAGTTAGTATTTCTTCAGTGATGGAT
                   ******** ****** * ***** ******************** **    ** ******

                          310       320       330       340       350       360
                   ....|....|....|....|....|....|....|....|....|....|....|....|
LfKCS3 original    ATTTTCTATGAGTTGAGAAAAGTTGATCCTTTGTGTGAGGTGGGTTGTGATGATTCTTCT
LfKCS3 modified    ATTTTCTATGAGTTGAGAAAGGTTGATCCACTTTGCGAAGTGGGTTGTGATGATAGTTCT
                   ******************** ********  * ** ** ***************  ****

                          370       380       390       400       410       420
                   ....|....|....|....|....|....|....|....|....|....|....|....|
LfKCS3 original    CTTGAGTTTATGAGGAAGGTTTTAGAACGTTCAGGTTTAGGTGATGAGACTTATGTTCCA
LfKCS3 modified    CTTGAGTTTATGAGAAAGGTTTTGGAGAGATCAGGTCTTGGAGATGAAACTTACGTTCCT
                   ************** ******** **  * ****** * ** ***** ***** ***** 

                          430       440       450       460       470       480
                   ....|....|....|....|....|....|....|....|....|....|....|....|
LfKCS3 original    CTTGGACTCCATCAAGTGCCACCTCAAAAGACTTTTGCAGCGATAAAGGACGAGACAGAG
LfKCS3 modified    CTTGGATTGCACCAAGTGCCACCTCAGAAAACTTTTGCTGCAATTAAGGATGAAACAGAG
                   ****** * ** ************** ** ******** ** ** ***** ** ******

                          490       500       510       520       530       540
                   ....|....|....|....|....|....|....|....|....|....|....|....|
LfKCS3 original    CAGGTAATCAAAGGTGCACTTGAGAATCTATTCGAGAACACAAAAGTAAACCCTAGAGAG
LfKCS3 modified    CAAGTTATTAAGGGTGCATTGGAAAATCTTTTCGAGAACACAAAGGTTAATCCTAGAGAG
                   ** ** ** ** ****** * ** ***** ************** ** ** *********

                          550       560       570       580       590       600
                   ....|....|....|....|....|....|....|....|....|....|....|....|
LfKCS3 original    ATTGGTATACTTGTGATAAACTCAAGCATGTTTAATCCAACACCTTCTCTATCAGCCATG
```

```
LfKCS3 modified   ATTGGTATTCTTGTTATTAATTCTTCTATGTTCAATCCAACACCTTCACTTAGTGCTATG
                  ******** ***** ** ** **    ***** ************** **    ** ***

                         610       620       630       640       650       660
                  ....|....|....|....|....|....|....|....|....|....|....|....|
LfKCS3 original   GTTATTAATACTTTCAAACTCCGAAGTAACATCAAAAGCTTTAATCTTGGAGGAATGGGT
LfKCS3 modified   GTTATCAATACCTTTAAATTGAGGTCAAACATTAAGAGTTTCAATCTTGGTGGAATGGGT
                  ***** ***** ** *** *  *    ***** ** ** ** ******** *********

                         670       680       690       700       710       720
                  ....|....|....|....|....|....|....|....|....|....|....|....|
LfKCS3 original   TGTAGTGCTGGTGTAATCGCCATTGATCTAGCTAAAGACTTGTTGCATGTTCATAAAAAC
LfKCS3 modified   TGCAGTGCTGGAGTTATCGCAATTGATTTGGCTAAAGATCTTTTGCATGTTCACAAGAAC
                  ** ******** ** ***** ****** * ********  * *********** ** ***

                         730       740       750       760       770       780
                  ....|....|....|....|....|....|....|....|....|....|....|....|
LfKCS3 original   ACTTATGCTCTTGTAATAAGCACTGAGAACATCACCATAACCGCTTATGCTGGCGAAAAT
LfKCS3 modified   ACCTATGCTCTTGTGATTTCAACTGAGAACATCACAATCACCGCTTACGCAGGTGAAAAC
                  ** *********** **    ************** ** ******** ** ** *****

                         790       800       810       820       830       840
                  ....|....|....|....|....|....|....|....|....|....|....|....|
LfKCS3 original   CGATCCATGAATGTTAGTAATTGCTTGTTCCGAATAGGCGGGGCCGCGATTTTGCTCTCT
LfKCS3 modified   AGATCTATGAATGTTTCAAACTGCCTTTTCAGGATCGGTGGAGCTGCAATACTTTTGAGT
                   **** *********   ** *** * *** * ** ** ** ** ** **  *  *   *

                         850       860       870       880       890       900
                  ....|....|....|....|....|....|....|....|....|....|....|....|
LfKCS3 original   AATAAGCCAAGAGATAAGAGAAGGTCTAAGTACAAGCTAGCTCACACTGTTCGAACACAA
LfKCS3 modified   AATAAGCCAAGAGATAAGAGAAGGTCTAAGTATAAGCTTGCACATACTGTTAGAACTCAC
                  ******************************** ***** ** ** ****** **** **

                         910       920       930       940       950       960
                  ....|....|....|....|....|....|....|....|....|....|....|....|
LfKCS3 original   ACAGGAGCTGATGACATGTCTTATAGATGTGTGCAACAAGAAGAAGATGAGATGGGTAAA
LfKCS3 modified   ACAGGAGCTGATGATATGTCTTACAGATGTGTGCAACAGGAAGAGGATGAAATGGGTAAA
                  ************** ******** ************** ***** ***** *********

                         970       980       990      1000      1010      1020
                  ....|....|....|....|....|....|....|....|....|....|....|....|
LfKCS3 original   GTAGGAGTTCGTCTCTCAAAGGACATAACTACTGTCGCGGGCACAGCAGTTAAGAAGAAC
LfKCS3 modified   GTTGGAGTGAGGCTTTCTAAGGATATTACTACAGTTGCTGGTACTGCAGTGAAGAAAAAT
                  ** *****  * ** ** ***** ** ***** ** ** ** ** ***** ***** **

                        1030      1040      1050      1060      1070      1080
                  ....|....|....|....|....|....|....|....|....|....|....|....|
LfKCS3 original   ATATCAACATTAGGTCCACTGATTCTTCCTTTAAGCGAAAAGCTTCTTTATTTCGTTTCC
LfKCS3 modified   ATCTCAACTCTTGGACCACTTATTTTGCCTCTTTCTGAGAAGCTTTTGTACTTCGTTTCA
                  ** *****  * ** ***** *** * *** *    ** ****** * ** ********

                        1090      1100      1110      1120      1130      1140
                  ....|....|....|....|....|....|....|....|....|....|....|....|
LfKCS3 original   TTCATCGCGAAGAAACTTTTGAAGGAGAAGATCAAGAACTATTACGTCCCGGATCTTAAG
LfKCS3 modified   TTCATAGCTAAGAAACTTTTGAAGGAAAAAATTAAGAACTACTACGTGCCAGATTTGAAG
                  ***** ** ***************** ** ** ******** ***** ** *** * ***

                        1150      1160      1170      1180      1190      1200
                  ....|....|....|....|....|....|....|....|....|....|....|....|
LfKCS3 original   CTAGCTATCAATCATTTTTGTATCCATGCTGGTGGAAGAGGTGTGTTAGATGTGTTGGAA
LfKCS3 modified   CTTGCTATTAATCATTTCTGCATCCACGCTGGTGGAAGAGGTGTTTTGGATGTGCTTGAG
```

```
                   ** ***** ******** ** ***** ***************** ** ****** * **

                         1210      1220      1230      1240      1250      1260
                   ....|....|....|....|....|....|....|....|....|....|....|....|
LfKCS3 original    AAGAACTTAAGGCTATCACCAATTGATGTAGAAGCATCTAGATCGACTTTACATAGATTT
LfKCS3 modified    AAGAATTTGAGGCTTTCTCCTATTGATGTTGAAGCTTCTAGATCAACATTGCATAGGTTC
                   ***** ** ***** ** ** ******** ***** ******** ** ** ***** **

                         1270      1280      1290      1300      1310      1320
                   ....|....|....|....|....|....|....|....|....|....|....|....|
LfKCS3 original    GGGAATACTTCTTCGAGTTCGATTTGGTATGAGTTGGCTTATATTGAAGCTAAAGGAAGG
LfKCS3 modified    GGAAACACCTCTTCAAGTTCTATATGGTATGAACTTGCATACATCGAGGCTAAGGGTAGG
                   ** ** ** ***** ***** ** ********  * ** ** ** ** ***** ** ***

                         1330      1340      1350      1360      1370      1380
                   ....|....|....|....|....|....|....|....|....|....|....|....|
LfKCS3 original    ATGAAGAAAGGTGATAAAGCTTGGCAAATTGCTTTAGGGTCAGGGTTTAAGTGTAATAGT
LfKCS3 modified    ATGAAGAAAGGAGATAAGGCATGGCAGATTGCTTTGGGTTCAGGTTTTAAGTGTAATAGT
                   *********** ***** ** ***** ******** ** ***** ***************

                         1390      1400      1410      1420      1430      1440
                   ....|....|....|....|....|....|....|....|....|....|....|....|
LfKCS3 original    GCTGTTTGGGTTGCTTTACGTAATGTTAAAGCTTCGGCTAATAGTCCTTGGGAACATTGT
LfKCS3 modified    GCAGTTTGGGTGGCTCTTAGAAACGTTAAGGCTAGTGCAAATTCTCCTTGGGAGCATTGC
                   ** ******** *** *  * ** ***** ***   ** ***  ********* *****

                         1450      1460      1470      1480      1490
                   ....|....|....|....|....|....|....|....|....|....|.
LfKCS3 original    ATTGATAGGTATCCTGTTCCTGATACTTGTGTAGAAAATGGTAAGTCTTAG
LfKCS3 modified    ATTGATAGGTATCCAGTTCCTGATACATGTGTGGAAAACGGAAAGTCTTGA
                   ************** *********** ***** ***** ** *******
```

Figure 3

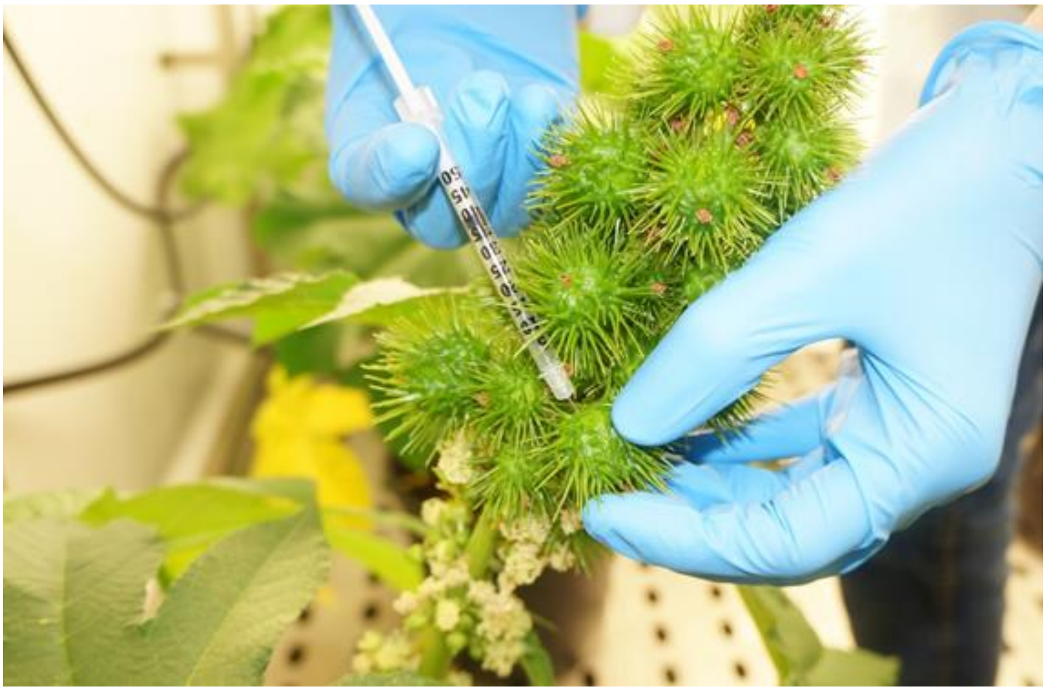

Injection of Agrobacterium in developing castor fruit

Figure 4

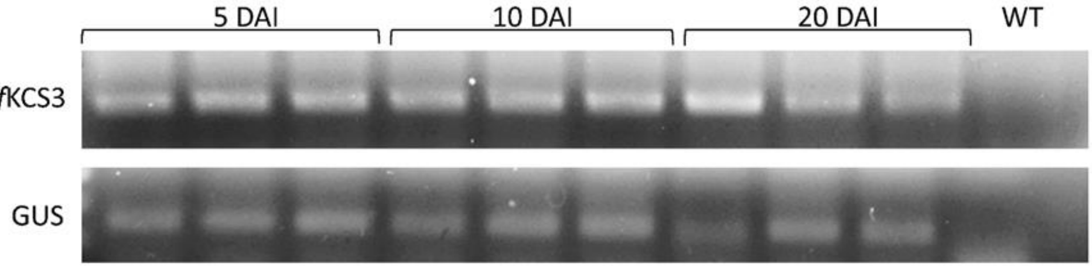

PCR transformation control in castor endosperm transiently transformed with *Lesquerella fedlery* KCS.
